# Genetic Detection of Dobrava/Belgrade Virus, Bulgaria

**DOI:** 10.3201/eid1702.101275

**Published:** 2011-02

**Authors:** Anna Papa, Iva Christova

**Affiliations:** Author affiliations: Medical School, Aristotle University of Thessaloniki, Thessaloniki, Greece (A. Papa);; National Centre of Infectious and Parasitic Diseases, Sofia, Bulgaria (I. Christova)

**Keywords:** Hantavirus, hemorrhagic fever, renal, pulmonary syndrome, rodents, viruses, Bulgaria, letter

**To the Editor:** Hantaviruses (family *Bunyaviridae*, genus *Hantavirus*) cause 2 clinical syndromes in humans: hemorrhagic fever with renal syndrome (HFRS) in the Old World and hantavirus pulmonary syndrome in the New World (*1*). Humans are infected by inhaling the excreta of infected rodents. Persons at increased risk for infection are farmers, loggers/forest workers, and soldiers.

Bulgaria is a country in southeastern Europe with 8 million inhabitants. Two types of hemorrhagic fevers are endemic to Bulgaria: Crimean-Congo hemorrhagic fever and HFRS. Both diseases have been subject to mandatory reporting since 1953. During the past decade, 36 cases of HFRS have been reported, mainly in the areas of the Balkan and Rila-Pirin-Rodopa mountain ranges in southwestern Bulgaria. Diagnosis was based on clinical symptoms and serologic test results. We report 3 HFRS cases, 2 of which were fatal. Apart from serologic diagnosis, genetic detection of hantaviruses was also achieved, resulting in gaining insight into the genetic relationships of hantavirus sequences from Bulgaria with respective sequences retrieved in neighboring countries.

On September 2, 2009, high fever, chills, headache, and myalgia developed in a 21-year-old man who lived in Simitli town (Blagoevgrad Province, southwestern Bulgaria). Five days after symptom onset, he was admitted to the regional hospital of Blagoevgrad. His condition rapidly deteriorated. Clinical signs were pharyngeal hyperemia, oliguria, and febrile toxic syndrome. The patient became hypotensive, reporting abdominal pain in the liver and spleen. Laboratory findings showed the following: leukocyte count 11.8 × 10^9^ cells/L, hematocrit 51%, blood hemoglobin 161 g/L, platelet count 10 × 10^9^ cells/L, aspartate aminotransferase (AST) 118 U/L, alanine aminotransferase (ALT) 89 U/L, urea 26.4 mmol/L, and creatinine 501 µmol/L. An echograph showed enlarged kidneys, liver, spleen, and pancreas, and abdominal and bilateral pleural effusions. Urine analysis disclosed proteinuria and microscopic hematuria. The patient was admitted with acute renal failure and multiple organ insufficiency. Despite multiple blood transfusions and hemodialysis, he died 14 days after hospitalization.

On April 9, 2010, a 54-year-old man, a resident of Kirkovo village (Kardjali Province, southern Bulgaria), was admitted to the regional hospital in Kardjali City with a 7-day history of fever, weakness, and myalgia in the lower extremities and a 4-day history of abdominal pain and diarrhea. At admission, physical examination showed skin petechiae, subconjunctival and gingival hemorrhages, and oliguria. Laboratory findings showed the following: leukocyte count of 23 × 10^9^ cells/L, platelet count of 50 × 10^9^ cells/L, AST 96 U/L, ALT 167 U/L, urea 58.7 mmol/L, and creatinine 1,033 µmol/L. Urea and creatinine levels continued to rise. Proteinurua and hematuria were present. After 3 sessions of hemodyalisis, the patient gradually improved, and he was discharged without sequelae.

On May 7, 2010, a 28-year-old man, a resident of Smilyan village, (Smolyan Province, southern Bulgaria) was admitted to the Infectious Diseases Clinic in Smolyan Regional Hospital with a 4-day history of fever, vomiting, and diarrhea. Physical examination on admission showed skin petechiae and gingival hemorrhages. Laboratory findings showed the following: leukocyte count of 6 × 10^9^ cells/L, platelet count of 50 × 10^9^ cells/L, urea 10.5 mmol/L, creatinine 230 mmol/L, AST 1697 U/L, and ALT 1,119 U/L. Proteinuria and hematuria were present. The patient became anuric and underwent hemodialysis. On May 9, the patient died.

Serum samples from these 3 patients were tested for immunoglobulin (Ig) G and IgM against Hantaan virus (HTNV) and Puumala virus by ELISA (Progen, Biotechnik GmbH, Heidelberg, Germany). High titers of HTNV IgM were detected in all 3 patients; in 1 patient HTNV IgG was also detected; antibodies against Puumala virus were not detected. Thus, a HTNV-like infection was suggested.

Viral RNA was extracted from the earliest available serum sample, and a 1-step SYBR Green real time reverse transcription–PCR (RT-PCR) (Bio-Rad, Hercules, CA, USA) (*2*) and 2 nested RT-PCRs amplifying partial small (S) and medium (M) RNA segments were applied (*3**,**4*). Dobrava/Belgrade virus (DOBV) RNA was detected by RT-PCR. Sequencing and phylogenetic analysis of the nested RT-PCR products showed that the causative agent in all 3 cases was DOBV (Figure).

**Figure:**
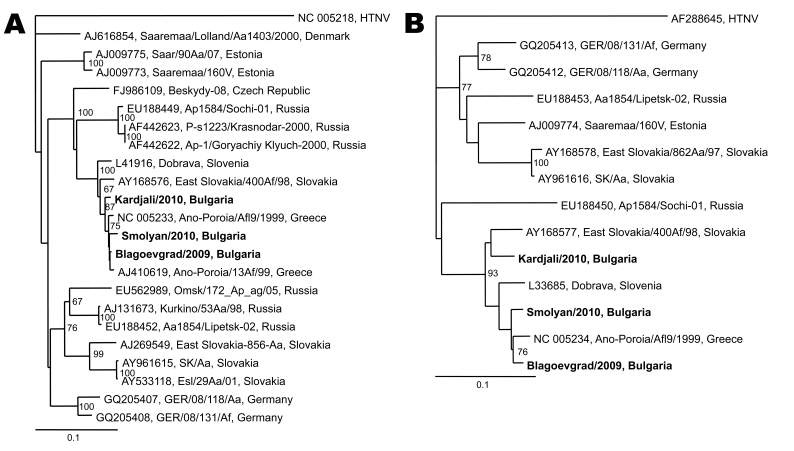
Phylogenetic trees based on a 560-bp fragment of the small RNA segment (A) and a 224-bp fragment of the medium RNA segment (B) of hantaviruses. Hantaan virus (HTNV) was used as the outgroup. The numbers at the nodes indicate percentage bootstrap replicates of 100; values <60% are not shown. Horizontal distances are proportional to the nucleotide differences. Sequences in the tree are indicated as GenBank accession number, strain name, country. Strains from this study are shown in **boldface**. Scale bars indicate 10% nucleotide sequence divergence.

Sequences were submitted to GenBank under accession nos. HQ174468–HQ174473. Bulgarian sequences cluster with respective sequences retrieved from *Apodemus flavicollis* mouse tissues or from HFRS cases from central and southeastern Europe. Briefly, the closest genetic strains in S and M RNA segments are strains isolated from *A. flavicollis* mice in northeastern Greece, near the border with Bulgaria (*5*). The genetic difference at nucleotide level among the Bulgarian strains is 1.2%–2.1% and 2.2%–7.4% in the S and M segments, respectively.

HFRS is endemic to the Balkan Peninsula. Severe HFRS cases caused by DOBV have been reported in Greece (*4**–**6*), Slovenia (*7**,**8*), Serbia and Montenegro (*9*), the Czech Republic (*3*), and Hungary (*10*). Our results confirm that DOBV also circulates in Bulgaria and causes severe HFRS cases; thus, clinicians have to include HFRS in differential diagnosis of febrile cases accompanied by acute nephropathy. Further studies on patients and small mammals in Bulgaria will elucidate the hantavirus epidemiology in this Balkan region.

## References

[1] Jonsson CB, Figueiredo LT, Vapalahti O. A global perspective on hantavirus ecology, epidemiology, and disease. Clin Microbiol Rev. 2010;23:412–41. 10.1128/CMR.00062-09 20375360 PMC2863364

[2] Aitichou M, Saleh SS, McElroy AK, Schmaljohn C, Ibrahim MS. Identification of Dobrava, Hantaan, Seoul, and Puumala viruses by one-step real-time RT-PCR. J Virol Methods. 2005;124:21–6. 10.1016/j.jviromet.2004.10.004 15664046

[3] Papa A, Zelena H, Barnetova D, Petrousova L. Genetic detection of Dobrava/Belgrade virus in a Czech patient with haemorrhagic fever with renal syndrome. Clin Microbiol Infect. 2010;16:1187–90. 10.1111/j.1469-0691.2009.03075.x 19832712

[4] Papa A, Johnson AM, Stockton PC, Bowen MD, Spiropoulou CF, Alexiou-Daniel S, Retrospective serological and genetic study of the distribution of hantaviruses in Greece. J Med Virol. 1998;55:321–7. 10.1002/(SICI)1096-9071(199808)55:4<321::AID-JMV11>3.0.CO;2-H 9661842

[5] Papa A, Nemirov K, Henttonen H, Niemimaa J, Antoniadis A, Vaheri A, Isolation of Dobrava virus from *Apodemus flavicollis* in Greece. J Clin Microbiol. 2001;39:2291–3. 10.1128/JCM.39.6.2291-2293.2001 11376073 PMC88127

[6] Papa A, Antoniadis A. Hantavirus infections in Greece–an update. Eur J Epidemiol. 2001;17:189–94. 10.1023/A:1017987104363 11599695

[7] Avsic-Zupanc T, Petrovec M, Furlan P, Kaps R, Elgh F, Lundkvist A. Hemorrhagic fever with renal syndrome in the Dolenjska region of Slovenia—a 10-year survey. Clin Infect Dis. 1999;28:860–5. 10.1086/515185 10825051

[8] Saksida A, Duh D, Korva M, Avsic-Zupanc T. Dobrava virus RNA load in patients who have hemorrhagic fever with renal syndrome. J Infect Dis. 2008;197:681–5. 10.1086/527485 18269319

[9] Papa A, Bojovic B, Antoniadis A. Hantaviruses in Serbia and Montenegro. Emerg Infect Dis. 2006;12:1015–8. 16707066 10.3201/eid1206.051564 PMC3373030

[10] Jakab F, Sebok J, Ferenczi E, Horvath G, Szucs G. First detection of Dobrava hantavirus from a patient with severe haemorrhagic fever with renal syndrome by SYBR Green-based real time RT-PCR. Scand J Infect Dis. 2007;39:902–6. 10.1080/00365540701387072 17852891

